# Reliable transgene-independent method for determining *Sleeping Beauty *transposon copy numbers

**DOI:** 10.1186/1759-8753-2-5

**Published:** 2011-03-03

**Authors:** Orsolya Kolacsek, Virág Krízsik, Anita Schamberger, Zsuzsa Erdei, Ágota Apáti, György Várady, Lajos Mátés, Zsuzsanna Izsvák, Zoltán Ivics, Balázs Sarkadi, Tamás I Orbán

**Affiliations:** 1 Membrane Research Group of the Hungarian Academy of Sciences, Semmelweis University and National Blood Center, Budapest, Hungary; 2 Mobile DNA Group, Max-Delbrück Center for Molecular Medicine, Berlin, Germany; 3 Department of Human Genetics, University of Debrecen, Debrecen, Hungary

## Abstract

**Background:**

The transposon-based gene delivery technique is emerging as a method of choice for gene therapy. The *Sleeping Beauty *(SB) system has become one of the most favored methods, because of its efficiency and its random integration profile. Copy-number determination of the delivered transgene is a crucial task, but a universal method for measuring this is lacking. In this paper, we show that a real-time quantitative PCR-based, transgene-independent (qPCR-TI) method is able to determine SB transposon copy numbers regardless of the genetic cargo.

**Results:**

We designed a specific PCR assay to amplify the left inverted repeat-direct repeat region of SB, and used it together with the single-copy control gene *RPPH1 *and a reference genomic DNA of known copy number. The qPCR-TI method allowed rapid and accurate determination of SB transposon copy numbers in various cell types, including human embryonic stem cells. We also found that this sensitive, rapid, highly reproducible and non-radioactive method is just as accurate and reliable as the widely used blotting techniques or the transposon display method. Because the assay is specific for the inverted repeat region of the transposon, it could be used in any system where the SB transposon is the genetic vehicle.

**Conclusions:**

We have developed a transgene-independent method to determine copy numbers of transgenes delivered by the SB transposon system. The technique is based on a quantitative real-time PCR detection method, offering a sensitive, non-radioactive, rapid and accurate approach, which has a potential to be used for gene therapy.

## Background

Transposon-based systems have become the method of choice for gene delivery, and their applications as potential genetic vehicles are receiving great interest [1-3]. In recent years, the *Sleeping Beauty *(SB) transposon has been emerging as the most favorable delivery system, because of its random integration profile and the lack of similar transposon-like elements in the human genome, which significantly minimizes the risk often represented by viral-based methods [4-6]. Owing to its advantageous characteristics, SB is the first transposon-based system to be used in a clinical trial for a hematologic malignancy [7]. Recently, a novel hyperactive version of the originally reconstituted SB transposase was developed [8], which, apart from making the system more favorable than other widely used non-viral methods, further substantiates its applicability as a mutagenic tool to perform genetic analyses, similar to the transposon-based systems in *D. melanogaster *and *C. elegans *[9,10]. Although already possessing clear advantages, rigorous characterization of the SB system still remains to be carried out to set up standard methods concerning its applicability. One of the important issues in setting up gene-therapy guidelines or genome-wide mutagenesis protocols is that of copy-number determination in stable clones [11-13].

Various technical methods have been developed to determine transgene copy numbers after gene delivery, including Southern blotting and the specific PCR-based transposon display method [14,15]. In most cases, these are performed using radioactively labeled probes; although fluorescent labeling can also be used, its threshold detection levels are generally lower. Depending on the transgene used, other techniques such as *in situ *hybridization quantification of fluorescent marker proteins such as green fluorescent protein (GFP) can also be employed [16]. Although these methods are widely accepted and used, they are usually laborious and require specific chemicals and equipment. In addition, these detection methods are often limited to the measurement of a specific transgene, and lengthy pilot experiments are often required to determine the exact measurements needed to accurately quantify a newly arising gene of interest within a particular delivery system [17-19].

During this study, we aimed to develop an accurate method for quantifying SB transposon copy numbers, independent of the transgene sequence. We term this the real-time quantitative PCR-based, transgene-independent (qPCR-TI) method. It can be used for any SB-based gene delivery experiments without *a priori *optimization of the protocol.

To establish this method, we used specific probe sets designed for the left and right inverted repeat-direct repeat (IRDR) regions, which are the recognition motifs of the transposase and therefore required for any SB transposition reaction [20]. As an internal control for normalization, a probe for the *RPPH1 *gene, the H1 RNA subunit of the RNaseP enzyme complex, was used. This gene is a widely accepted one-copy gene of the haploid human genome [21]. Comparing this system with the radioactive transposon display and Southern/dot blotting techniques, we provide evidence that using the IRDR-L specific probe set in comparative 2^-ΔΔCt ^measurements can reliably and accurately quantify SB transposon copy numbers in various cell lines, regardless of the transgene used. Apart from being sensitive, accurate and rapid, this real-time PCR-based quantification method also offers a powerful non-radioactive technique as an alternative against other standard methods.

## Results and Discussion

**Figure 1:**
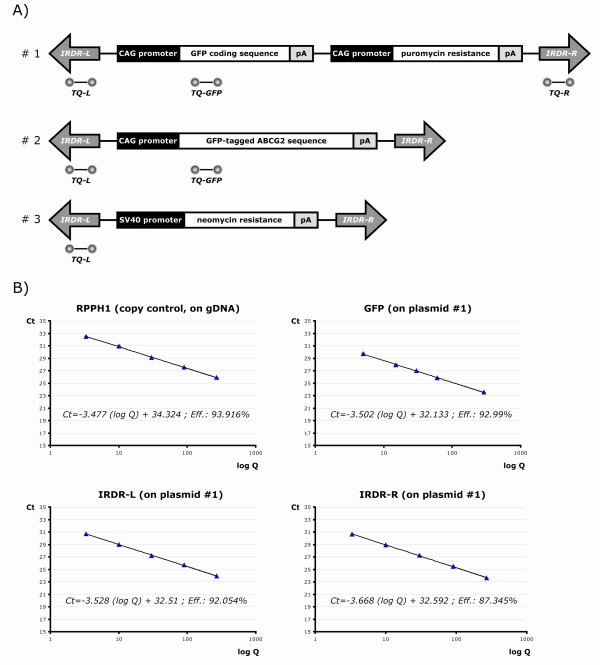
**Real-time PCR assay designed for different transposon and transgene regions**. **(A) **Structure of the used SB transposons with asymmetric IRDRs [22]. For each construct, the TaqMan^® ^assays (TQ) used for copy-number determination are indicated. Sequences are not drawn to scale. IRDR-L/-R = inverted repeat-direct repeat left/right regions; pA = SV40 polyadenylation signals. **(B) **Efficiencies of the real-time assays determined by standard curves. For all assays, a dilution series was prepared from pooled genomic DNA samples from clones containing integrated transposon 1. The efficiency of the IRDR-R TaqMan^® ^assay was notably lower than that of the others (<90%).

The exact and rapid quantification of transgene copy numbers is often required for gene-delivery experiments. As we generally use the SB transposon system in our laboratory, we aimed to develop a real-time PCR-based technique that would be transgene-independent, specific for the transposon regions and therefore widely applicable. To optimize the qPCR-TI method, we began with clones of HEK-293 cells with SB transposons carrying two transcription units expressing GFP and the puromycin-resistance gene, which are both under the control of the CAG promoter (Figure 1A). This transgene setup allowed generation of clones with various copy numbers by either fluorescence-activated cell sorting (FACS) or antibiotic selection. Specific TaqMan^® ^(Applied Biosystems, Foster City, CA, USA) assays were designed for the two IRDR motifs of the SB transposon and for the GFP sequence (Figure 1A). The widely applicable SB transposon version used throughout this study has two asymmetric IRDR regions ('left' and 'right' [22]). In most transposon flanking sequences, the two IRDR regions are repeat-rich DNA sequences, which makes PCR primer design relatively difficult. Moreover, the left and the right IRDRs are very similar to each other, which further increases the difficulty of designing specific assays for them. Nevertheless, we could still develop specific assays for each; neither of the IRDR-L nor the IRDR-R probe set gave signals in the exclusive presence of the other template (data not shown).

As the first (and simplest) approach, absolute quantification of DNA samples was performed using plasmid dilution series complemented with transposon-free non-specific genomic (g)DNA. However, the difficulties of determining the exact nucleic-acid concentration of very dilute samples and the differences in purity between samples made it necessary to abandon absolute quantification, and to include an internal copy control to overcome these problems with relative quantification. The *RPPH1 *gene, the H1 RNA subunit of the RNaseP enzyme complex, was chosen as this is a widely-accepted one copy gene of the haploid human genome [21] (http://www.ncbi.nlm.nih.gov/ieb/research/acembly/index.html). However, the assay efficiency for the IRDR-R region differed significantly from that of the others, including the *RPPH1 *endogenous control assay. Various conditions for the IRDR-R set were tried, and although template concentration seemed to be a crucial factor, the widely accepted template range of 10 to 40 ng still produced efficiency values that were significantly lower than those of the other assays (<90%) (Figure 1B). Sequence constraints originating from the similarity to IRDR-L hindered us designing other specific assays with different combinations of primers and probes in this short (228 bp) and repeat-rich region. Therefore, if this assay were to be included for measurement, the relative standard curve method would be the only acceptable quantification method, as it is the most suitable to compare reactions with suboptimal PCR efficiency. Apart from the setting up of standard curves (for both the transposon-specific assays and the *RPPH1 *endogenous control), relative quantification also requires the use of a calibrator (a reference sample with a known copy number, preferably '1') to ensure the precision of quantification.

**Figure 2:**
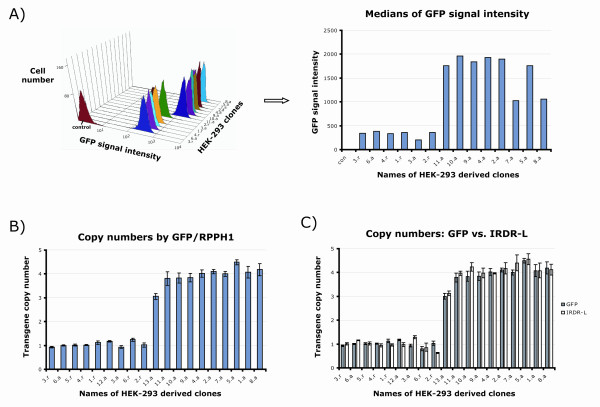
**Comparing copy-number determination by green fluorescent protein (GFP) or transposon-specific real-time PCR**. **(A) **Fluorescence-activated cell sorting (FACS) analysis of different HEK-293 derived clones expressing GFP. Higher fluorescent intensities indicate higher copy numbers, although signals can vary because of integration position effects and/or transgene silencing. The control sample shows the autofluorescence detected in non-transfected HEK-293 cells. **(B) **Copy numbers determined by transgene (GFP) specific real-time PCR assay normalized to the level of one copy control *RPPH1*; clones analyzed by FACS (A) and other clones established subsequently were examined. Various clones with low GFP expression level were determined to have one integrated transposon copy, whereas the majority with higher GFP fluorescence was found to have four transposon copies. In the case of clone 5.a, further analysis revealed that it was not a clone but rather a mixture of clones with an average copy number around 4.5. **(C) **Comparison of two techniques. The copy values determined by the transgene independent TaqMan^® ^assay for the IRDR-L sequence correlated well with the GFP-based copy numbers. Clone 2.r originated from random integration, so the transposon repeat sequence might not be intact, and the partial presence of IRDR-L could result in a lower signal, therefore this clone was not included among the controls for later experiments. a = clones obtained from active transposition; r = clones obtained from random integration (from transfection with the mutant transposase). For copy numbers, values are means ± SEM of at least three independent measurements.

In the search for a potential calibrator sample, generated clones were screened by FACS for the lowest possible GFP signal, assuming that clones with one copy number should be among those samples (the signal could also vary because of positional effects of different integration sites). Although the CAG promoter we used is known to be less prone to silencing [23-25], we had to make sure the lowest fluorescent signals were also associated with the lowest real-time signals when normalized to the *RPPH1 *level, in order to exclude the potential presence of silenced copies. Using the GFP TaqMan^® ^assay, several clones with one integrated transposon copy and numerous others with three or four copies were found (Figure 2A,B). Using the IRDR-L set, very similar copy numbers could be calculated using the relative standard curve method (Figure 2C), whereas the IRDR-R TaqMan^® ^set gave unreliable results, mainly due to the problems discussed earlier (Additional file 1). Because the assays for *RPPH1*, GFP and the transposon IRDR-L had very similar efficiency values (Figure 1B), we also tried another approach, calculating the copy numbers in the examined clones by the comparative Ct (2^-ΔΔCt^) method in the same experiments. The results based on GFP or IRDR-L were in agreement with each other and with the results of the relative standard curve method. Moreover, technical errors could be further decreased by using a pool of gDNA samples with known copy number as a reference. We therefore concluded that once we left out the specific but less efficient assay for the IRDR-R region, the comparative Ct method could be used for reliable and precise transposon copy-number determination using the IRDR-L TaqMan^® ^assay. Abandoning the relative standard curve method also allowed inclusion of more samples in one reaction plate, as no more dilution series with several parallels were required.

**Figure 3:**
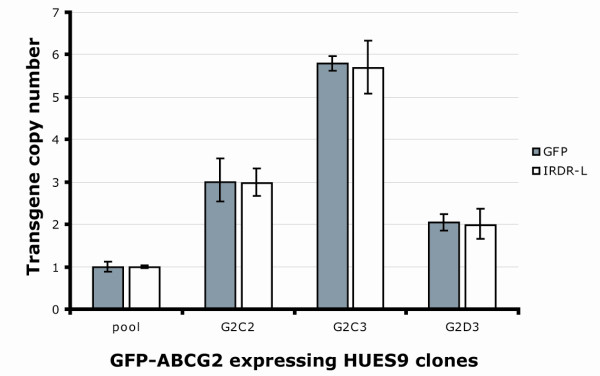
**Copy-number determinations of green fluorescent protein (GFP)-ABCG2 expressing HUES9 clones**. The sample 'pool' indicates the equimolar mixture of gDNA samples from the first four single-copy clones on Figure 2C. Later examination of the G2C3 line indicates that it is not derived from a clone but rather from a mixture of cells with five and six transposon copies. Values are means ± SEM of at least three independent measurements.

To test the qPCR-TI method on other samples, we examined clones of the HUES9 human embryonic stem cell line expressing the GFP-tagged ABCG2 transporter [26] generated by the SB transposon system. Again, the GFP and the IRDR-L TaqMan^® ^assays could be compared with each other (Figure 1A, transposon 2). As a general assay setup, the *RPPH1 *control and reference samples (pool of clones with known copy numbers) were used. As shown in Figure 3, the 2^-ΔΔCt ^method produced the same copy numbers, using either of the probe sets. These experiments therefore supported the use of the IRDR-L repeat specific assay for transposon copy-number determination, as it gave the same results as the assay specific for the carried internal transgene.

To compare our transgene-independent quantification approach with other techniques, we measured copy numbers of clones generated from HeLa cells by transposons containing a neomycin-resistance (neoR) gene (Figure 1A, transposon 3). Such clones were ideal for comparison because of the different transgene sequences and because their copy numbers were also determined by the Southern/dot blotting techniques or the transposon display method [5]. Several clones were tested, and the qPCR-TI method gave the same copy numbers as determined by the other radioactive methods (Table 1). For higher (>5) copy-number clones, the qPCR-TI method was also reasonably accurate, with occasional low relative-error margins (≤9%). The slight differences in some cases could be due to the inaccuracy of the standard methods for this range [14,15]. In addition, it has been suggested that precise values of very high copy numbers are more reliably measured by dot blot rather than transposon display methods. We found that the copy number of clone 4 determined by the dot-blot technique correlated well with data produced by the qPCR-TI. For low copy-number clones, only one clone (2/2 of neoR; see Table 1) did not give identical results with the different techniques. A difference of one copy number here clearly represents a higher percentage error margin, but this error might be related to the difference in integration sites in that particular clone (see discussion below). Taken together, the results of the neoR transposon clones indicated that the qPCR-TI technique is just as sensitive and accurate as the other widely used methods.

**Table 1:** Comparing the qPCR-TI method with other standard techniques

Clone name	Methods	Copy numbers	
		
		By standard methods	**By qPCR-TI**^**a**^
***Transposons carrying the neomycin resistance gene***			

2/1	Transposon display/Southern blotting	8 to 10	8

2/2	Transposon display/Southern blotting	3	4

2/3	Transposon display/Southern blotting	10 to 12	10

2/9	Transposon display/Southern blotting	1	1

1	Transposon display/Southern blotting	12 to 13	13

4	Dot blot	52	50

5	Transposon display/Southern blotting	15	15

6	Transposon display/Southern blotting	12	11

7	Transposon display/Southern blotting	1	1

8	Transposon display/Southern blotting	2	2

9	Transposon display/Southern blotting	1	1

***Transposons carrying the amaxaGFP transgene***			

A3	Splinkerette PCR/inverse PCR	2	2

A4	Splinkerette PCR/inverse PCR	4	4

A5	Splinkerette PCR/inverse PCR	4	4.5^b^

A6	Splinkerette PCR/inverse PCR	2	2

B1	Splinkerette PCR/inverse PCR	1	2

B3	Splinkerette PCR/inverse PCR	3	3

B5	Splinkerette PCR/inverse PCR	2	2

^a^Quantitative PCR, transgene independent.

^b^For sample A5 from the amaxa green fluorescent clones, real-time PCR measurement indicated that it is more likely to be a mixed population of cells rather than a single clone.

A further proof of principle was given by the determination of the transposon copy numbers in HUES9 clones previously generated using another sequentially distinct transgene. In those experiments, the amaxaGFP (a special fluorescent protein from a *Pontellina *copepod species, http://www.lonzabio.com) was carried by the transposon to generate clones of an embryonic stem-cell line, and the transposon integration sites were determined by the splinkerette PCR and the inverse PCR methods [27]. Based on these integration assays, copy numbers were estimated to be one to six in various clones, although all integrated copies may not be reliably detected by these methods because of the different flanking genomic sequences. When using the qPCR-TI method for several clones using the IRDR-L assay, the measured transposon copies were almost always the same as those previously claimed on the basis of the different proven integration sites (Table 1). One exception here was clone B1, where qPCR-TI gave a result one copy higher, similarly to the 2/2 neoR clone. A difference of one copy number here again undoubtedly represents a higher discrepancy with higher percentage error margin. However, because all the other low copy-number clones gave identical results with the various techniques, the two outliers might represent the lower sensitivity of the standard methods due to the dependence of transgene-integration sites [15]. These comparisons lead us to the conclusion that the qPCR-TI method provides reliable results for different SB transposon constructs, thereby being a consistent transgene-independent copy-number quantification method.

Using the experiments described above, the newly developed transgene-independent method for determining SB transposon copy numbers was validated: (i) it provided the same results as the assays specific for the carried transgene sequence and (ii) it could also reliably replace widely used standard radioactive techniques. The TaqMan^® ^assay designed for the IRDR-L region of the transposon provides the basis for transgene independence as it is present in all SB constructs. In fact, 'symmetric' SB transposons with two IRDR-L (but not two IRDR-R) flanking sequences are functional [28], and the qPCR-TI method is also applicable to such constructs (with an obvious correction factor of 0.5). We found evidence that the PCR efficiency of this probe set is similar to the *RPPH1 *single-copy control, so reliable quantification can be performed using the comparative 2^-ΔΔCt ^method. To ensure precise and rapid quantification, reference samples (calibrators) with known copy numbers are also included, preferably a pool of gDNAs from different clones, to minimize discrepancies resulting from different transgenic sampling techniques and purities. The method could also be extended to other non-human gDNA samples; however, a suitable and validated single-copy reference gene control must always be used.

Another technical point that should be considered is the transposition-independent, random integration of the transgene. Because this is a stochastic process, it could possibly lead to the integration of the carried transcription unit without the transposon IRDR sequences. In such cases, the qPCR-TI method clearly underestimates transgene copy numbers, as it only detects copies resulted from *bona fide *transposition. As a general rule, we always include control experiments with gene delivery using the mutant transposase to estimate the level of random integration [20]. According to previous experiments, this phenomenon is generally very rare when using the new hyperactive SB100x transposase, but its extent can vary between different cell lines. Nevertheless, if such random background integration increases significantly, it may be necessary to measure the copy numbers of the transgene itself in the samples generated with the active transposase.

## Conclusions

We have developed a sensitive and reliable real-time PCR-based (qPCR-TI) method for measuring SB transposon copy numbers. When compared with widely used standard methods, such as various blotting techniques or transposon display, it proved to be just as accurate as those other methods, while also offering a faster and non-radioactive method. However, the real advantage of this method is the transgene independence, which makes it applicable for any scientists working with *Sleeping Beauty *transposon constructs. Therefore, we believe that qPCR-TI could become the method of choice for gene therapy and general gene-delivery applications.

## Methods

### Cell-culture maintenance and creation of clones

Human embryonic kidney cells (HEK-293) were cultured in Dulbecco's modified Eagle's medium (DMEM) supplemented with 10% fetal calf serum, 1% L-glutamine and 1% penicillin/streptomycin (Invitrogen, Carlsbad, CA, USA). Transfected cell populations were first enriched for transgene expression by flow cytometry (see below). Subsequently, cell clones were created by serial dilutions in 96-well plates. Selected clones were further analyzed by flow cytometry and harvested for genomic DNA isolation (see below). The HUES9 embryonic stem-cell line (originally provided by Dr. Douglas Melton, Harvard University, USA) was maintained essentially as described previously [29], using cells from passage 35. To create transgene-expressing HUES9 clones, we used our previously developed method for human embryonic stem-cell lines [27].

### Transfection and transposition

HEK-293 and HUES9 cells were transfected using a transfection reagent (FuGENE^® ^6; Roche Applied Science, Rotkreuz, Switzerland) in accordance with the manufacturer's instructions. The transfection mix contained 1 μg of a given transposon plasmid (Figure 1A) and 100 ng of the hyperactive SB100x *Sleeping Beauty *transposase, in a 10:1 ratio to minimize the overproduction inhibition phenomenon [5,8]. To visualize the random integration background, a control transfection with the inactive DDE motif mutant of the transposase was carried out, using the same experimental setup [20].

### Flow cytometry

GFP-expressing cells were analyzed by a flow cytometer (FACSCalibur; Becton-Dickinson, San Jose, CA, USA) with Cellquest-Pro analysis software (Becton-Dickinson). Mock-transfected cells were used as labeling controls, and propidium iodide or 7-aminoactinomycin D staining was used to exclude non-viable cells. To select and clone cells expressing GFP, a fluorescence based cell sorter (FACSAria High Speed Cell Sorter; Becton-Dickinson) was used in accordance with the manufacturer's instructions.

### Genomic DNA isolation, transposon display and Southern/dot blotting

After treatment with trypsin, cells were separated by centrifugation and washed with 1 × phosphate-buffered saline. After careful removal of the liquid supernatant, the dry cell pellets were stored at -80°C until further processing. Genomic DNAs were isolated from the cells by standard phenol-chloroform extraction after cell lysis and proteinase K digestion. DNA samples were quantified with a spectrophotometer (GeneQuant II; Pharmacia Biotech, Piscataway, NJ, USA). Transposon display and Southern-/dot-blotting techniques were performed essentially as described previously [5,14].

### Quantitative real-time PCR

Reactions were performed on a real-time PCR platform (StepOne™ or StepOnePlus™; Applied Biosystems, Foster City, CA, USA) in accordance with the manufacturer's instructions. The gDNA samples (30 ng each) were run in triplicate, in singleplex reactions with a final volume of 20 μl using TaqMan^® ^chemistry. All primers and probes were designed by Primer Express software (version 3.0; Applied Biosystems), and probes were labeled with 5'-FAM and 3'-nonfluorescent (minor groove binding) quencher molecules. Sequences for the TaqMan^® ^assays are given in Table 2. Final concentrations of primers and probes were 250 and 900 nM, respectively. Data were analyzed by StepOne software (version 2.1; Applied Biosystems).

**Table 2:** Primers and probes used for quantitative real-time PCR.

Primer/probe name	Sequence 5'→3'
*RPPH1*	

Forward	AGCTGAGTGCGTCCTGTCACT

Reverse	TCTGGCCCTAGTCTCAGACCTT

Probe	CACTCCCATGTCCC

GFP	

Forward	GAGCGCACCATCTTCTTCAAG

Reverse	TGTCGCCCTCGAACTTCAC

Probe	ACGACGGCAACTACA

IRDR-L	

Forward	CTCGTTTTTCAACTACTCCACAAATTTCT

Reverse	GTGTCATGCACAAAGTAGATGTCCTA

Probe	CTGACTTGCCAAAACT

IRDR-R	

Forward	GCTGAAATGAATCATTCTCTCTACTATTATTCTGA

Reverse	AATTCCCTGTCTTAGGTCAGTTAGGA

Probe	TCACCACTTTATTTTAAGAATGTG

## Competing interests

The authors declare that they have no competing interests.

## Authors' contributions

OK established the HEK clone; OK and VK optimized the real-time PCR and performed copy-number measurements; AS, ZE and ÁA established the HUES9 clones; GV helped in FACS measurements; LM measured copy numbers in HeLa clones; ZsI and ZI gave technical help and advices with the SB transposon work; BS provided financial support and discussed the data; and TIO designed the overall strategy, analyzed the data and wrote the paper.

## Supplementary Material

Additional file 1 **Supplementary Figure 1: Comparison of the IRDR-R assay with the GFP specific real-time PCR method**. Selected HEK-293 clones were examined for transposon copy numbers in parallel by the accepted green fluorescent protein (GFP) specific assay and the assay specific for *Sleeping Beauty *(SB) inverse repeat-direct repeat, right (IRDR)-R. In contrast to the IRDR, left (IRDR-L) real-time assay, the IRDR-R specific assay failed to reproduce previously determined copy numbers consistently (see Figure 2C). For this particular experiment, 30 ng genomic (g)DNA was used for the reaction. Although different starting gDNA concentrations (higher than the recommended range of 10 to 40 ng) improved the reproducibility of the IRDR-R assay, it still did not reach the reliability level of the GFP or the IRDR-L assays. Click here for file
