# Correction: Reliable transgene-independent method for determining sleeping beauty transposon copy numbers

**DOI:** 10.1186/1759-8753-4-11

**Published:** 2013-03-15

**Authors:** Orsolya Kolacsek, Virág Krízsik, Anita Schamberger, Zsuzsa Erdei, Ágota Apáti, György Várady, Lajos Mátés, Zsuzsanna Izsvák, Zoltán Ivics, Balázs Sarkadi, Tamás I Orbán

**Affiliations:** 1 Membrane Research Group of the Hungarian Academy of Sciences, Semmelweis University and National Blood Center, Budapest, Hungary; 2 Mobile DNA Group, Max-Delbrück Center for Molecular Medicine, Berlin, Germany; 3 University of Debrecen, Debrecen, Hungary

## Correction

After the publication of our article [1], the authors noticed two typing errors in the ’Methods’ part of the original article:

– in Table two, the correct sequence of *RPPH1* / Probe is 2 nucleotides longer at the 3’ end, so it is: CACTCCCATGTCCCTT ;

– in the penultimate sentence of the 'Quantitative real-time PCR' section, the concentrations of the primers and the probe were switched so the correct sentence should be: "Final concentrations of primers and probes were 900 and 250 nM, respectively."

The authors apologize for these unintended mistakes in the text.

## Competing interests

The authors declare that they have no competing interests.

## Authors’ contributions

OK established the HEK clones, OK and VK optimized the real-time PCR and performed copy number measurements, AS, ZE and ÁA established the HUES9 clones, GV helped in FACS measurements, LM measured copy numbers in HeLa clones, ZsI and ZI gave technical help and advices with the SB transposon work, BS provided financial support and discussed the data and TIO designed the overall strategy, analyzed the data and wrote the paper. All authors read and approved the final manuscript.
