# Supplementary figures and images for: Reliable transgene-independent method for determining Sleeping Beauty transposon copy numbers
Source: Mob DNA. 2011 Mar 3;2:5. doi: 10.1186/1759-8753-2-5 (PMC3060107; doi:10.1186/1759-8753-2-5)

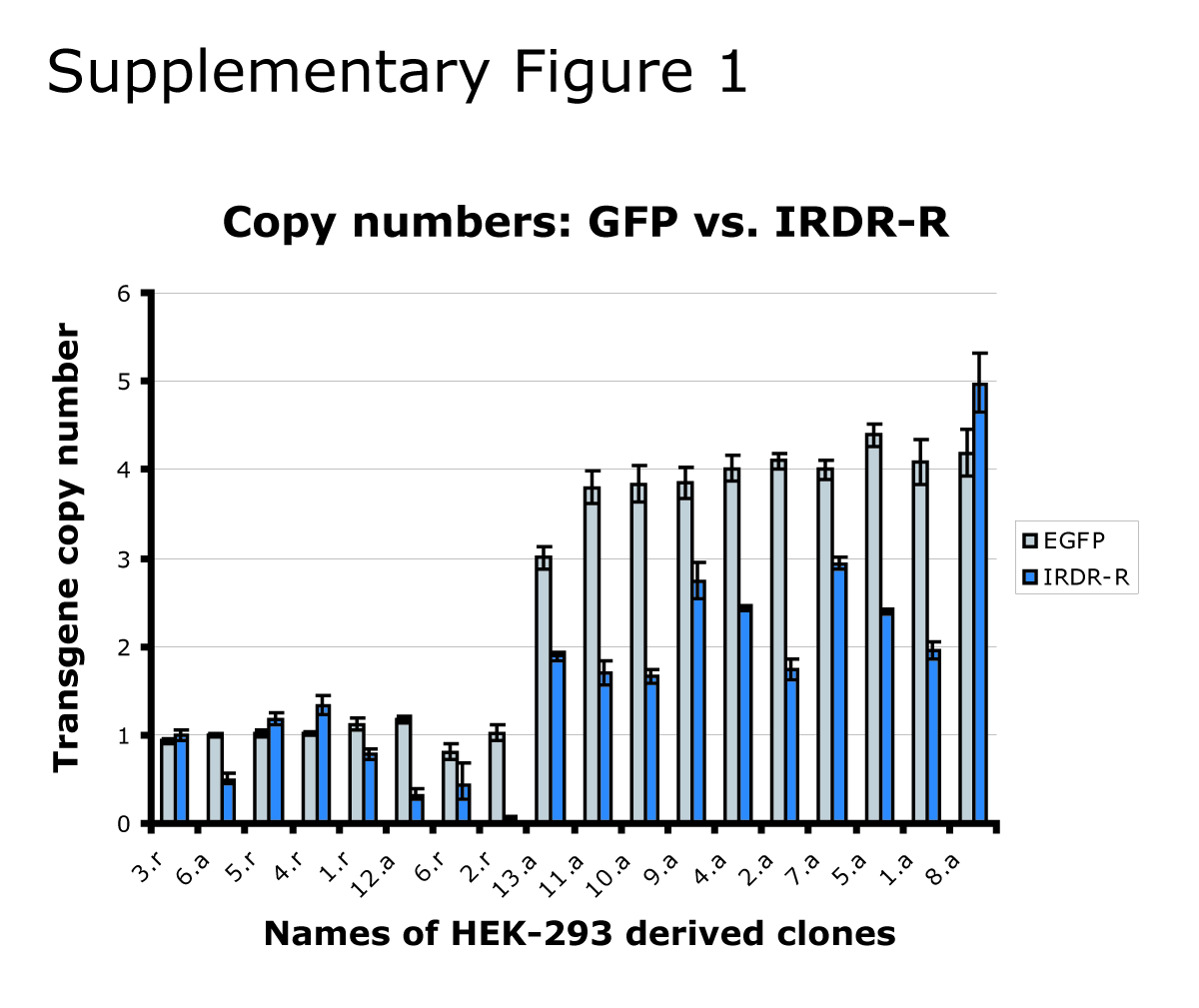

Supplement: Additional file 1 — Supplementary Figure 1: Comparison of the IRDR-R assay with the GFP specific real-time PCR method. Selected HEK-293 clones were examined for transposon copy numbers in parallel by the accepted green fluorescent protein (GFP) specific assay and the assay specific for Sleeping Beauty (SB) inverse repeat-direct repeat, right (IRDR)-R. In contrast to the IRDR, left (IRDR-L) real-time assay, the IRDR-R specific assay failed to reproduce previously determined copy numbers consistently (see Figure 2C). For this particular experiment, 30 ng genomic (g)DNA was used for the reaction. Although different starting gDNA concentrations (higher than the recommended range of 10 to 40 ng) improved the reproducibility of the IRDR-R assay, it still did not reach the reliability level of the GFP or the IRDR-L assays. [file 1759-8753-2-5-S1.TIFF]
